# Enhancing Credibility of Chemical Safety Studies: Emerging Consensus on Key Assessment Criteria

**DOI:** 10.1289/ehp.1002737

**Published:** 2010-12-15

**Authors:** James W. Conrad, Richard A. Becker

**Affiliations:** 1 Conrad Law and Policy Counsel, Washington, DC, USA; 2 American Chemistry Council, Washington, DC, USA

**Keywords:** chemical safety, credibility, industry funding, regulatory science, reliability, scientific integrity

## Abstract

**Objectives:**

We examined the extent to which consensus exists on the criteria that should be used for assessing the credibility of a scientific work, regardless of its funding source, and explored how these criteria might be implemented.

**Data sources:**

Three publications, all presented at a session of the 2009 annual meeting of the Society for Risk Analysis, have proposed a range of criteria for evaluating the credibility of scientific studies. At least two other similar sets of criteria have recently been proposed elsewhere.

**Data extraction/synthesis:**

In this article we review these criteria, highlight the commonalities among them, and integrate them into a list of 10 criteria. We also discuss issues inherent in any attempt to implement the criteria systematically.

**Conclusions:**

Recommendations by many scientists and policy experts converge on a finite list of criteria for assessing the credibility of a scientific study without regard to funding source. These criteria should be formalized through a consensus process or a governmental initiative that includes discussion and pilot application of a system for reproducibly implementing them. Formal establishment of such a system should enable the debate regarding chemical studies to move beyond funding issues and focus on scientific merit.

Federal agencies such as the Food and Drug Administration (FDA), National Toxicology Program (NTP), National Institute of Occupational Safety and Health (NIOSH), and National Institute of Environmental Health Sciences (NIEHS) devote substantial resources to evaluating chemical hazards. However, chemical product manufacturers have conducted the great bulk of toxicological testing and research used in regulatory safety assessments. For substances whose regulation requires preapproval (e.g., pharmaceuticals, food additives, and pesticides), regulatory frameworks require companies to conduct specific tests [Federal Food, Drug, and Cosmetic Act (FFDCA) 1938; Federal Insecticide, Fungicide, and Rodenticide (FIFRA) of 1972]. The U.S. Environmental Protection Agency (EPA) also has the power, by rule, order, or negotiated outcome, to direct companies to test chemicals they manufacture or process (or propose to manufacture or process) [Toxic Substances Control Act (TSCA) 1976]. In addition, companies routinely evaluate potential hazards and risks of their products to assure a safe workplace, for product stewardship, and to limit potential liability. As a result, industry-conducted or -funded research provides the bulk of the science relevant to assessing chemical safety, and this is not likely to change (Barden et al. 2006).

For almost as long as industry has been conducting research involving chemicals, skeptics have challenged the credibility of that work. They have described intentional generation of false or misleading data (Hirschhorn 2000) and research that seems directed to increasing doubt about health effects (Michaels 2008). Critics have argued that industry-supported work has employed methods, animal strains, or other test features that tend to miss or underestimate adverse effects (Myers et al. 2009). Commonly, no more specific criticism is leveled than that the results of the work tend to support the use, rather than restriction, of the chemical (Kissinger and Rust 2008). News and social media increasingly imply that industry support of scientific work is alone sufficient to invalidate it (Popken 2008). Even though the source of funding has been asserted to be a “less significant” cause of publication bias than other causes (e.g., academic pressure to publish) (DeMaria 2004; Fanelli 2010), industry support suffices for many to vitiate the credibility of scientific work.

The volume of industry-supported studies of chemicals is burgeoning now with the European Union’s implementation of its Registration, Evaluation, Authorisation and Restriction of Chemical substances (REACH) system (Hartung and Rovida 2009), and an amended TSCA in the United States could drive additional chemical testing, even if REACH data are considered for TSCA purposes (Safe Chemicals Act of 2011). But how will massive increases in the quantity of such data improve public confidence in the U.S. chemical regulatory system if industry-supported work is perceived as inherently dubious? Absent significant action to address that perception, we seem inexorably headed toward an outcome in which landmark legislation and substantial investments in research and testing produce little or no improvement in public confidence in either the safety of chemical products or the effectiveness of the system designed to assure it.

## A Necessary Solution: Consensus Criteria for Assessing Scientific Credibility

The crux of any solution to this problem of legitimacy in chemical evaluation is a means for assessing the trustworthiness of a study regardless of its funding source. A widely accepted set of objective criteria for assessing credibility, applicable by any interested person, is needed.

As we document here, there is already wide agreement on such criteria. Three sets of criteria proposed in 2009 by independent groups of experts, as well as two similar sets published recently, demonstrate a remarkable degree of convergence among them. This convergence augurs well for the prospect of a formalized system of credibility assessment, which could arise from a consensus process, a regulatory initiative, or both. To facilitate such an effort, we conclude our review by discussing issues that any such systemization effort would necessarily have to address.

## Credibility Distinguished from Reliability

Conventionally, the ultimate measure of a scientific work’s validity is how broadly and confidently its conclusions become accepted in the relevant field, which in turn derives from the extent to which its findings are replicated and extended. However, establishing such validity, especially for a novel experimental finding, can take years, and what constitutes replication or extension may be disputed for some time.

As a result, the policy-making community has evolved a variety of short-term proxies for evaluating the merits of a scientific work. The best known of these is Klimisch et al.’s criteria-based system for evaluating a study’s “reliability” for use in hazard or risk assessment (Klimisch et al. 1996). This system “is the most widely used system” of its sort (Dor et al. 2009), and the European Commission has supported development of a software-based tool (the “ToxRTool”) to extend and operationalize it (Schneider et al. 2009).

However, reliability alone is not a sufficient condition. Equally necessary is a proxy that addresses the credibility of a given work, that is, the likelihood that it faithfully reflects what was observed, is not the product of unconscious bias or intentional manipulation, and is thus believable. Such a proxy should be applicable to all sources of funding, and across all affiliations of investigators, because questions can also arise about the credibility of research by scientists funded by government agencies or nonprofit organizations. Without broad agreement on objective means for determining the credibility of research and testing, public confidence in regulatory evaluations and product stewardship programs will not improve. To facilitate progress, we propose Klimisch-like criteria for assessing the credibility of studies in light of any funding source or affiliation.

## Sources and Methodology

The genesis of this article was a session at the annual meeting of the Society for Risk Analysis (SRA) in December 2009 at which speakers presented three strikingly similar sets of criteria for evaluating scientific credibility:

- David Goldston presented a Bipartisan Policy Center (BPC) report titled “Improving the Use of Science in Regulatory Policy” (BPC 2009). This report represents the work of 13 distinguished academic, business, and nonprofit representatives, many of whom have held senior scientific or policy positions within government. It includes consensus recommendations on four topics, one of which is a discussion of “basic principles” for how federal agencies should filter and evaluate “studies relevant to regulatory policy” (BPC 2009).
- Eric Hentges and Sanford Miller presented a report of a panel of 14 other academic and industry experts organized under the aegis of the International Life Sciences Institute (ILSI). The report, published in the *American Journal of Clinical Nutrition* (Rowe et al. 2009), presents “guiding principles” for “health, nutrition and food safety science.”
- One of the authors of this review (J.W.C.) presented a slightly updated version of an earlier article (Henry and Conrad 2008) that identified “standards and practices currently being used [that] assist in judging the quality of research and testing . . . regardless of why the work was conducted” (Conrad 2009).

Each of the criteria presented either *a*) increases confidence that the sponsor or experimenter did not shape or skew the results or interpretation of an experiment; or *b*) enables others to assess independently whether such shaping or skewing occurred.

Further confirming our convergence thesis, two similar sets of credibility criteria have been published by the International Agency for Research on Cancer (IARC) and the Federation of American Societies for Experimental Biology (FASEB). The IARC (2008) document is oriented toward a broader purpose (a code of good scientific practice), and the FASEB document (Brockway and Furcht 2006) toward biomedical research, but many of their elements are comparable to the criteria discussed at the SRA meeting and thus are also discussed here.

Here, we attempt to advance the foregoing literature by demonstrating the degree of commonality among the five sets of criteria, synthesizing them into a discrete set, and exploring issues that would have to be addressed in any effort to systematize application of the criteria.

Here, we have intentionally omitted two potential criteria. First, we do not propose that funding by industry (or any other source that could be perceived to have a biasing effect) should be itself a criterion of lack of credibility, because that would be antithetical to science: “The observing scientist is only an accessory in the acquisition of scientific evidence, where the facts are asked to first speak for themselves” (Borgert 2007). A question about the source of funding merely serves to trigger application of the criteria. Second, we do not propose considering whether a third party administered the funding of scientific work (e.g., Cohen et al. 2009) because such a mechanism seems too costly and complicated to be applied broadly under REACH or TSCA. There are clearly situations where such third-party administration of research efforts has been fruitful, such as the Health Effects Institute (2010) program on the health effects of air pollution. Although such an approach perhaps could be employed to investigate basic research areas of a select set of commodity chemicals, it is difficult to envision for regulatory scientific studies within a modernized TSCA that would need to deal with the approximately 15,000 chemicals that the U.S. EPA estimates are currently in commerce (U.S. EPA 2010a). However, full consideration should be given to use of independent third-party entities to evaluate and verify regulatory testing systems within companies or consortia for scientific integrity, to provide input into complex study designs or testing protocols of substances of high social concern, and to conduct peer review of major testing reports, especially studies that are not published in scientific journals.

## Proposed Credibility Criteria

The 10 proposed criteria are shown in Table 1, along with information on the extent to which a given criterion is contained in the five publications (or the presentation) on which this article is based. In the table, the check mark (✓) indicates complete or substantial adoption, the qualified check mark (~✓) indicates partial adoption, and the dash (—) indicates absence. The criteria are ranked by how broadly they are contained within the five underlying publications and are ranked in rough order of importance within those groups.

Below we explain these criteria more fully, highlighting considerations that warrant further discussion regarding how each criterion might be applied. We frankly acknowledge, as did Klimisch et al. (1996), that judgment is inherent in that process. This is not fatal to the overall concept, but rather another reason why a consensus or governmental process should be undertaken to promote discussion of the criteria and how they could be systematized.

### Criterion 1: Whether the principal investigator (PI) has fully disclosed sources of funding and other “competing interests.”

To control for the prospect that a study’s funding source might lead to bias, the most essential element is disclosure of funding sources. There may be legitimate circumstances in which such disclosure is not possible; for example, a historic study may not disclose funding sources. Still, as stated by the BPC (2009), “agencies and scientific advisory committees should be wary of studies when it is unclear who funded the study or whether the principal investigator(s) had any conflicts of interest.”

Notably, this criterion is not limited to funding from “industry”; it anticipates that PIs will disclose all sources of financial support received, whether by grant or by contract, in support of the relevant work. This is the perspective of the BPC (2009), IARC (2008), and Henry and Conrad (2008).

Biasing financial interests may also arise from sources beyond those funding the research. Concerns about the biasing effect of investigators’ financial interests have, for example, led the U.S. Department of Health and Human Services (DHHS) and the National Science Foundation (NSF) to issue investigator conflict of interest policies (DHHS 1995; NSF 2005); both require grant applicants to disclose “significant financial interests” of the PIs or their immediate family that “would reasonably appear to be affected” by the research proposed to be funded. Most journals similarly require prospective authors to disclose all “competing interests” [e.g., *Environmental Health Perspectives* (*EHP*) 2010]. Consistently, the five underlying articles go beyond disclosure of funding sources to call for “full signed disclosure of all financial interests” (Rowe et al. 2009), disclosure of “any conflicts of interest” (BPC 2009), or “acknowledge[ment of] all forms of external support” (IARC 2008), or at least note the growth of “competing financial interests policies” (Henry and Conrad 2008). The FASEB guidelines (Brockway and Furcht 2006) are particularly explicit about the range of relationships that should be disclosed by investigators, including funding, consulting, equity ownership, and any other “significant financial interests.”

As the BPC (2009) noted, substantial thought should be devoted to determining what constitutes a conflicting or competing interest and to balancing the goal of promoting disclosure against investigators’ legitimate interests in privacy and minimizing burdens. The BPC and Henry and Conrad (2008) both recommended the National Academies (2003) policy on this topic as a good model. The BPC (2009) also noted the disagreement within its own panel regarding whether disclosure should be limited to current interests. (The DHHS and NSF policies apply only to current interests, whereas *EHP*’s policy and the BPC require 3- and 2-year look-backs, respectively.) There is thus much work to be done on this criterion by any future consensus or governmental effort to formalize this approach. But there is wide agreement on the core elements and their importance.

### Criterion 2: Whether the PI is legally guaranteed the right to a) publish the results of the study without prior sponsor approval, b) analyze and interpret the resulting data, and, where appropriate, c) control the study design

#### Right to publish

All five of the underlying sets of credibility criteria also agree that reliable science involves the PI, by written agreement with the funding source, having the right to publish the study results without prior sponsor approval. This substantially bolsters the work’s credibility because it gives the PI authorial control and prevents the sponsor from being able to conceal or selectively release results.

This criterion does not prevent the PI from agreeing to share a draft study protocol, progress report, or draft article or report with the sponsor, because *a*) the PI and the study sponsor may wish to consult about whether observed effects are reportable under applicable law; *b*) prepublication review could be necessary for the sponsor to secure intellectual property rights in the study subject (Brockway and Furcht 2006); and *c*) sponsor comment could result in a more accurate report (Henry and Conrad 2008). If prepublication review is anticipated, this criterion requires prior delineation of the permissible basis for review and applicable time limits. Such provisions would help to guard against unjustified deferral of publication that could be caused by extensive or repeated rounds of comments or by undue delay in the sponsor providing comments to the PI.

The ILSI report and IARC code went beyond guaranteeing the PI’s right to publish by requiring a written agreement that “obligat[es the PI] to attempt to publish the findings within some specified timeframe” (Rowe et al. 2009) or speaking of a “duty to publish” (IARC 2008). Further discussion of this criterion should specifically consider whether a publication obligation is appropriate for all types of scientific work (e.g., product testing) or where publication is not a goal of the PI. A system to implement this criterion might award extra “credit” where publication occurs.

#### Control of data analysis/interpretation

Henry and Conrad (2008) endorsed the practice of the American Chemistry Council’s Long-Range Research Initiative to give its PIs ownership of resulting data (American Chemistry Council 2009). They explained that doing so “lend[s] strength, objectivity, and credibility to the outcome of the research” because “when investigators own the data and the scientific information that they generate . . . they are in control of how those data will be evaluated, used and communicated” (Henry and Conrad 2008). No other set of criteria discussed here specifies that investigators must own data, presumably because sponsors often need to retain ownership of data for regulatory purposes or to preserve patent or other legal protections. On the other hand, a PI does not have to own data to be given independent control in analyzing it. The result is the same: the sponsor has no legal basis to prevent the PI from independently interpreting the data.

The ILSI report stated that investigators must be “guarantee[d] control of statistical analysis” of data (Rowe et al. 2009), and the FASEB guidelines stated that investigators “shall have access to, and be involved in, the analysis and/or interpretation of all data generated in the research” (Brockway and Furcht 2006). Going slightly beyond these formulations, the criterion we propose requires that the PI explicitly be given independent authority to analyze and interpret the study results.

#### Control of research design

The BPC (2009) and the ILSI (Rowe et al. 2009) specified that the PI should also retain control of the study design. Neither discussed the issue in any detail, which is unfortunate because there may well be circumstances where this requirement is not fully appropriate. Certainly, PI control of study design would seem to be of questionable applicability in cases where the design of the study is determined in advance by explicit regulatory agency direction. For example, FIFRA and TSCA require adherence to test guidelines that prescribe experimental study design elements (U.S. EPA 1989a, 1989b), and the Organization for Economic Cooperation and Development (OECD 1998) imposes similar requirements. Finally, the Animal Welfare Act (1966) required study designs involving animal models to be reviewed and approved in advance by an institutional animal care and use committee (IACUC). IACUC approval requires consideration of numerous study design elements, including justification of the animal model, number of animals, procedures for animal care, and approaches to monitor and address pain and distress.

The credibility of the study is certainly boosted where control of study design is given to the PI, and so the proposed criterion requires it where feasible. (The appropriateness of the chosen design is an important but separate issue, covered under criterion 4.)

### Criterion 3: Whether the investigator or sponsor has publicly released the research data or test method to allow others to review them and seek to replicate the analysis

Although it is important for PIs to have the legal right to release study results, it is equally important from a credibility perspective for the PI or the sponsor to release the underlying data and methods, so that others can evaluate how well the study supports the claimed conclusion (or some other alternative, plausible conclusion). The ability to interpret data or replicate results typically also requires knowledge of the research or test method employed. Hence, this criterion rewards prompt dissemination of data and methods by publication in the scientific literature, submission of the results to the government, or other similar public distribution.

This criterion is likely to be controversial because academic scientists have not traditionally released the actual data underlying their studies, at least initially. Scientific journal publications generally include only a summarization of experimental procedures and summary tables or figures; laboratory study records and raw data are typically retained by the PI. Nor do most academic investigators adhere to GLP requirements, which require government access to complete study records and data sets (FDA 2006; U.S. EPA 2010b). Often, academics intend to publish several articles from a data set and do not want to reduce the prospect or impact of later publications by releasing the data. Mindful of these sensitivities, the IARC (2008) code stated a principle of “full and contemporaneous documentation of research methods [and] data,” but it provided that the “principle[] in practice” requires only that “requests for use of the data should be encouraged, with due regard to the terms on which the data were obtained and stipulations that may have been made.” The FASEB guidelines (Brockway and Furcht 2006) adopted a similar approach: “Once a study is published, academic investigators should make reasonable efforts to provide data and materials to other investigators for replication purposes.”

In contrast, the “Shelby Amendment” (Omnibus Appropriations Act 1998) and Circular A-110 of the Office of Management and Budget (OMB 1999) require federal agencies to make publicly available, via the Freedom of Information Act (FOIA 1966), final research data generated by agency grantees that an agency cites in support of a rule or order. It seems only fair for privately funded work to be subject to the same disclosure requirement, at least when the persons conducting or funding it submit it to an agency. This is the position taken by the BPC (2009). [Henry and Conrad (2008) contended that agencies actually have that disclosure obligation whenever they rely on privately funded information, as a result of the Information Quality Act (IQA 2000) and the OMB IQA guidelines (OMB 2002).] Particular research may involve confidential business information [e.g., proprietary data or software that cannot be publicly disclosed without violating the intellectual property rights of the vendors or inventor (who may be the PI)]. FOIA protects such information, although due process or administrative law concerns may be triggered if an agency relies on nonpublic data.

Outside of the contexts where federal law requires disclosure of data, some form of compromise or accommodation on the topic may be appropriate. The ILSI report (Rowe et al. 2009) called for “guarantee[ing] accessibility to all data and control of statistical analysis by investigators and appropriate auditors/reviewers.” A more demanding standard might be to expect release of underlying data and methods by some reasonable period of time after initial publication. Alternatively, there may be ways the PI can describe the data or methods sufficiently to enable evaluation without revealing specific intellectual property.

Disclosing all results of a study, not just those that can be summarized in a journal article, permits regulatory agencies to independently evaluate the integrity of study data, verify the authors’ conclusions, and, if needed, analyze the results using alternative procedures. The space restrictions imposed by paper journals limit their ability to publish data and thus impede the use by agencies of studies reported solely in such journals. The Internet circumvents this limitation, however, and so the practice of scientific journals such as *EHP* of enabling investigators to post supplemental data online is bound to increase. Although such supplemental information is not fully equivalent to the complete data supplied for a GLP study, it likely will not be long before such data posting becomes the norm. Therefore, the scientific and regulatory communities need to accelerate development of standardized toxicological data sets that capture the information required to adequately describe a study for deposition into online databases (Fostel et al. 2007).

### Criterion 4: Whether the investigator conducted research that was designed objectively and reported factually, so that, according to accepted principles of scientific inquiry, the research design adequately tests an appropriately phrased hypothesis

This criterion, adapted from the ILSI report (Rowe et al. 2009), is self-evident. The credibility of studies assessing the safety of chemical products depends critically on the ability to have confidence in their procedures and the resulting data (Becker et al. 2009), and the most important procedural aspect may be that the research design not favor a particular outcome. This has become a hotly contested issue. For example, some argue that traditional toxicity testing methods that follow U.S. EPA or OECD test guidelines and GLP methods are “out of date” and “insensitive” and thus “incapable of detecting low-dose endocrine-disrupting effects” (Myers et al. 2009). On the other side, criticisms related to study design have been leveled at academic studies for using irrelevant exposure routes (NTP–Center for the Evaluation of Risks to Human Reproduction 2007).

#### Importance of agency test guidelines and GLPs

Henry and Conrad (2008) previously presented a detailed discussion of the GLP regulations that the U.S. EPA and FDA require experimenters to follow in conducting studies to be submitted under FIFRA, TSCA, and FFDCA (FDA 2006; U.S. EPA 1989a, 1989b). Henry and Conrad (2008) discussed how these rules require experimenters to *a*) document study conduct and results; *b*) create and use written standard operating procedures (SOPs); and *c*) follow agency-approved, validated study protocols. Maintenance of complete study records and data sets allows independent verification of critical study elements such as *a*) development and adherence to SOPs and protocols for each study component; *b*) dosing techniques; *c*) use of group sizes adequate to provide meaningful statistical analysis; *d*) analytical characterization of test and control substances with respect to identity, purity, and concentration; *e*) the veracity of recording of study measurements and data; and *f*) incorporation of quality control procedures and independent quality assurance reviews. For these reasons, the Klimisch et al. (1996) criteria award “the highest grade of reliability” to studies that follow “internationally accepted test guidelines . . . and the principles of . . . GLP,” as do systems following them (Dor et al. 2009; Küster et al. 2009).

The same features that render guideline-compliant GLP studies reliable also make them more credible because they enable one to say confidently that the PI followed the specified protocol, actually took the steps and measurements claimed to have been taken, and accurately reported the results. Four of the five underlying sets of criteria note this fact expressly (Henry and Conrad 2008), constitute a code of good scientific practice (IARC 2008), or call for PIs to adhere to such standards (Brockway and Furcht 2006; Rowe et al. 2009). Accordingly, we recommend that any implementation of this criterion judge studies meeting GLPs as more credible than those that do not.

This recommendation may draw criticism from scientists who do not follow standardized test guidelines and GLPs and would object to the additional costs and effort inherent in doing so. However, when such studies are submitted to or considered by agencies for policy purposes, it seems only appropriate to regard them as less credible than studies that follow test guidelines and GLPs, where applicable.

As Klimisch et al. (1996) recognized, there is an inherent dynamic tension between standardized and validated test methods and new, cutting-edge scientific methodologies that academic researchers develop and apply to evaluate new hypotheses. Both have important roles and provide valuable data. Options for establishing the requisite degree of confidence in new methods or novel findings include traditional structures for conducting method validation (e.g., the Interagency Coordinating Committee on the Validation of Alternative Methodologies) or using approaches such as evidence-based toxicology (Guzelian et al. 2005; Hoffmann and Hartung 2006) or those discussed by the National Research Council (2007).

#### Good epidemiology practices

For epidemiology studies, the guidelines for Good Epidemiological Practice (GEP) (International Epidemiological Association 2007) and the similar “Guidelines for Good Pharmacoepidemiology Practices” (GPP) provide “minimum practices and procedures that should be considered to help ensure the quality and integrity . . . and to provide adequate documentation of research methods and results” (International Society for Pharmacoepidemiology 2007). However, the extent to which epidemiological studies published in scientific journals meet such professional society best practices is unclear because authors do not typically include this information in publications.

GEP/GPPs require many of the same credibility-related features as GLPs: a detailed research protocol based on a stated hypothesis before the study starts; data collection, analysis, and storage procedures; and quality control measures. [GEP/GPPs also provide reliability-related guidance on reaching causal inferences derived from the Hill criteria (Hill 1965).] Although none of the underlying sets of criteria discuss GEP/GPPs, we believe that PIs of epidemiological studies should be encouraged to meet them and to so indicate in their articles. Future discussion of this criterion should consider judging epidemiology studies meeting GEP/GPPs as more credible than those that do not. This would also present an opportunity to discuss whether the model of an independent work group, similar to a pathology working group, could provide greater credibility for particularly influential epidemiological studies.

#### Involvement of outside experts in study design and choice of method

Another factor that justifies greater credibility in a study’s design or a new method is whether it was chosen (or that choice was reviewed) by outside experts. Thus, according to the IARC (2008) code, “The study design and choice of method . . . should have passed through a scientific review.” This factor will usually be satisfied in cases where government regulations or conditions specify the study design, because those requirements generally have been developed with external scientific review. Where external expert involvement is feasible, a study should be regarded as more credible when it occurred.

#### Conformance to the “Common Rule” is an additional index of credibility

The Common Rule protects the rights and welfare of human research subjects and governs human subjects research conducted, funded, or regulated by federal agencies (DHHS 1991). [Human subjects research not directly covered by the Common Rule must nonetheless conform to it in order to be considered by the U.S. EPA in connection with its pesticide program (U.S. EPA 2006).] Comparable guidelines also exist for research conducted outside the United States (e.g., World Medical Association 1964). A key element of the Common Rule is the use of an institutional review board (IRB) to review the proposed research for ethicality. Among the criteria that the IRB must apply is that any risks to the subjects be “reasonable in relation to anticipated benefits, if any, to subjects, and the importance of the knowledge that may reasonably be expected to result from the work” (DHHS 1991). In order to produce new knowledge, research has to be designed and conducted in ways that will produce reliable results; in particular, it must contain a significant and clearly articulated study question, and its design must be sufficiently rigorous to answer that question. Therefore, a human study’s conformance to the Common Rule or an international equivalent is another independent basis for concluding that this criterion is satisfied, and such a study should be judged more credible than a nonconforming study.

### Criterion 5: Whether the work was peer reviewed

Virtually all of the underlying publications recognized the centrality of peer review. Henry and Conrad (2008) observed that a “rigorous peer review is a key part of the foundation on which scientific excellence is achieved in all research programs.”The BPC (2009) stated that “papers in high impact, peer reviewed journals should be given great weight, and papers that have not been peer-reviewed should be treated with skepticism.” The ILSI report (Rowe et al. 2009) did not specify peer review as one of its guiding principles but declared that a “strong peer review system . . . is a mandatory prerequisite for these guidelines to be effective.” Likewise, the IARC (2008) code spoke of the “duty to publish . . . in peer-reviewed scientific publications.”

BPC (2009) also recognized that “the quality of peer review varies widely” and that agencies “need to extend their inquiry beyond simply ascertaining whether a paper was peer reviewed,” potentially to include “how the peer review was conducted.” (BPC also proposed ways to strengthen the peer review process.) Henry and Conrad (2008) emphasized the major differences between journal peer review and the “more demanding” reviews that OMB’s *Peer Review Bulletin* (OMB 2005) imposes on federal agencies regarding science they propose to rely on—particularly “highly influential scientific assessments.” The U.S. EPA’s Science Advisory Board, the NTP’s Board of Scientific Counselors, and similar government-administered panels exhibit a level of thorougness and transparency not characteristic of journal peer review when they peer review individual studies. (The *Peer Review Bulletin* does not require a panel considering a risk assessment or similar review of an integrated set of studies to review the underlying studies individually.) Accordingly, this criterion regards studies that have undergone governmental peer review pursuant to the *Peer Review Bulletin* as more credible than those that have not.

We decline to adopt the BPC’s (2009) proposed higher weight for “high impact” journals, given the uncertainty about where the line should be drawn between “high” and “low” impact and the prospect that this approach might import a degree of publication bias into the reliability assessment process.

Finally, additional discussion of this criterion should address the important role of histopathological evaluations in determining adverse effects in toxicological studies. For “pivotal general toxicology studies” the Society of Toxicologic Pathology (STP) recommended that a second pathologist independently review the slides to arrive at more solid decisions (STP 1991), and in some cases a formal pathology working group can be convened (Ward et al. 1995). The STP position papers on reporting pathological findings (Morton et al. 2006) and pathology peer review (Morton et al. 2010) provided up-to-date discussions of these important elements of GLP toxicology studies. Clearly, an independent pathology review, conducted in accordance with STP guidelines, increases the credibility of a toxicity study.

### Criterion 6: Whether, before its commencement, the study was included on a public registry of research intended for use in policy making

The BPC (2009) report highlighted the growth of public study registries, the best known of which is ClinicalTrials.gov, which Congress required the National Institutes of Health (NIH) to establish for clinical trials of certain pharmaceuticals (NIH 2010). Where such a registry exists, the BPC report recommended that agencies consider registered studies and “be wary of studies that met the criteria for the registry, but were not registered.”

One benefit of clinical trial registries is that sponsors are required to alert the world that they are initiating a study and cannot await the outcome before deciding whether to announce it. Another is that neither the sponsors nor the PI can redefine the purpose of a study to match what was found rather than what was initially hypothesized. A third is that such registries may promote the publication of negative results, a needed corrective to the increasingly recognized problem of publication bias and its tendency to produce overstatements of toxicity, efficacy, or other variables being assessed (DeMaria 2004; Sena 2010).

Although there do not yet appear to be formal registries for toxicological and epidemiological studies of the sort involved in chemical safety testing, there is no reason why an agency such as the U.S. EPA, or various private entities, could not establish one. The U.S. EPA has experience in implementing a publicly accessible database containing test plans and results from the High Production Volume Challenge program (U.S. EPA 2010b), and the NTP has long maintained a database that includes chemicals the organization is currently testing and substances that have completed testing (NTP 2010). Similarly, the Risk Information Exchange is an Internet database containing notifications about a variety of human health risk assessment projects in progress or recently completed (Alliance for Risk Assessment 2010). Thus, it appears reasonably feasible to establish such a registry for toxicological and epidemiological studies, open to all studies regardless of sponsor. The proposed criterion would regard a study as more credible if, once such a registry is established, the study had been timely registered.

### Criterion 7: Whether the PI’s remuneration was geared to a particular experimental outcome

Compensation contingent upon results would seem the very essence of a conflict between the interests of truth and the experimenter and the antithesis of credibility. Accordingly, ILSI listed as a guiding principle that investigators not accept such remuneration when conducting experimental studies (Rowe et al. 2009). Going further, we believe that an agreement to pay a PI on such a contingent basis renders a study inherently not credible and warrants its outright exclusion from agency consideration.

### Criterion 8: Whether a sponsor or a PI participated in an arrangement by which the sponsor would pay the PI to lend his or her name to a presentation or article actually drafted by someone else

Attention is increasingly being devoted to the phenomenon of parties seeking to leverage the reputations of esteemed scientists to boost the credibility of their product or activity. The ILSI principles included a prohibition on such reputation trading (Rowe et al. 2009). The DHHS has proposed to treat “paid authorship” as a “significant financial interest” (DHHS 2010) requiring disclosure and potential management under its investigator conflict of interest policy discussed above. Under this criterion, an article or presentation would be regarded as less credible if the nominal author had not in fact contributed substantially as an author or investigator but had accepted compensation to allow the actual author to use his or her name.

### Criterion 9: Whether a PI working under the auspices of a contract research organization (CRO) or other entity has maintained clarity between that entity and the PI’s academic or other affiliations

In the ILSI report, Rowe et al. (2009) noted that academic researchers who are also affiliated with other nonacademic institutions will sometimes contract to conduct research at the latter organization (i.e., the CRO) but, when publishing the results of that research, will list their academic affiliation (Rowe et al. 2009). As with the authorship phenomenon discussed above, this practice improperly trades on reputation. As with the prior criterion, if this practice occurs, the study should be regarded as less credible.

### Criterion 10: Whether the sponsoring organization employs systematic external review of research and testing programs to promote a culture of scientific integrity

Organizational dynamics do not spontaneously promote frank internal review. As a result, passive controls to ensure scientific integrity are now viewed as insufficient, and institutions are moving to proactive activities to educate and reinforce honesty, accuracy, and objectivity in research (DHHS 2006). At times, even internal procedures can become dated and less than optimally effective. The practice adopted by several major U.S. chemical products companies to counter such tendencies includes periodically asking highly respected independent experts to conduct systematic reviews of their research and testing programs (Conrad 2009).

Any organization—public or private—that sponsors research could benefit from such a process, and this criterion judges a study sponsored by an organization employing the process as more credible. Future deliberation should address how the process could be objectively and readily verified.

## The Next Step: A Formalized System of Credibility Assessment

There is already an encouraging convergence, across a wide range of experts and organizations, on the criteria that should be used to assess the credibility of scientific work. Establishing a similar consensus on a system to reproducibly apply the criteria will require much work, however, to resolve the following issues.

### Foreseeable controversies

Several of the criteria conflict with legitimate and strongly held values among relevant communities. Academic researchers in particular are likely to be concerned about criterion 3 (public release of data and methods), especially insofar as it provides greatest credit for full and timely release, and criterion 4 (research design) to the extent that it provides greatest credit for GLP-compliant studies. Protection of confidential business information will also be important to the private sector in connection with criterion 3.

### Missing criteria?

Because this article is a review, we are limited to the criteria contained in the five publications under review (although we do propose expanding some of them). An important next step would be to determine whether any other criteria warrant inclusion. For example, fair questions can be asked about whether sponsors are engaging the most knowledgeable investigators. Discourse on this issue should begin with the recognition that different purposes of scientific investigation (i.e., basic or applied research) can lead to different selection processes.

Academic basic research, research in its purest sense, is focused on increasing fundamental scientific knowledge. Basic research develops and evaluates new hypotheses, creates novel methods, and uses these to probe the limits of current understanding. Basic research is typically funded by research grants, which foster the scientific process by allowing considerable latitude in the research program.

In contrast, applied research tends to focus on defined aspects of a question and to employ well-established methods. Applied research is typically funded by contracts rather than grants, and these contracts characteristically specify in considerable detail the scope of the effort and the types of procedures to be used. This is especially true for toxicology studies required to comply with agency test guidelines. Although requests for proposals can be, and often are, used for applied research, it is also common practice for the private sector to employ focused solicitation of specific CROs or specific individual PIs. Sponsors may elect to contract with a specific CRO or PI that they feel has qualifications, expertise, and a track record of meeting regulatory requirements for study design, conduct, and reporting. Such an approach can lead to concern that industry may be selecting scientists whose previous work has concurred with an industry position. However, concerns about the process of selecting investigators can be assuaged by the credibility criteria discussed above. Thus, applied research should not be viewed as less credible when it is conducted through a focused solicitation.

### Qualitative or quantitative?

The next issue to be explored in considering how to establish a system of credibility assessment is whether the criteria should simply be used as a list of factors to be considered, or whether studies should be “scored” numerically against them. The former, qualitative approach has considerable precedent, including the Hill (1965) considerations. Nonetheless, such an approach leaves enormous discretion to users and is unlikely to be reproducible by different individuals or panels. Also, some criteria seem intuitively more important than others (indeed, in this article we rank them in part on that basis) and a numerical approach would enable consistent weighting of criteria. We thus favor exploration of a system that would score studies against the criteria, with specific numbers of points or credits available under each criterion. Such an approach must yet grapple with several issues, each of which will require thoughtful debate.

- **Relative weighting.** It is one thing to agree that some criteria are more important than others but quite another to agree on how much weight each deserves.
- **Binary or continuum?** A criterion might simply be satisfied or not—criterion 6 (inclusion on a registry) is a good example. Others, however, might be applicable in a graded way that could result in assignment of more points as the criterion was more fully satisfied. This approach would seem most applicable to criteria 4 (research design and conduct) and 5 (peer review).
- **Should any criteria be grounds for disqualification?** A study that fails to meet a criterion might simply receive no credit. Alternatively, the criterion might be deemed so fundamental that failure to satisfy it renders a study inherently not credible, as we have proposed for criterion 7 (compensation geared to experimental outcome).
- **Normalization.** Many aspects of the criteria will not always be applicable to a specific study; for example, a PI has little freedom to design an agency-required test guideline/GLP study, and no widely established registry of toxicity studies yet exists. Other aspects may be the basis for purely “bonus” points on the theory that they are always potentially applicable (e.g., whether a study was subject to governmental peer review). The design of a scoring system would require careful thought about how to take account of these aspects.
- **Who can use the system?** Because government agencies are charged with determining the safety of chemicals, the proposed criteria are principally intended for those agencies. There is no question, however, that broad use by a range of stakeholders is inevitable—the strongest argument for developing a credibility scoring system that is readily useful and reproducible.

### Next steps

For a credibility assessment system itself to have credibility, the foregoing issues need to be addressed by a group of stakeholders with diverse perspectives. This could occur through some sort of formal consensus process, a governmental initiative, or both. In either case, it will be crucial to reach out to potentially affected parties and to actively seek practical input on specific questions and examples.

It particular, it would be wise to conduct a pilot of any proposed credibility assessment system. The pilot could *a*) identify a range of studies that are relevant to a given safety assessment, including industry-funded guideline/GLP studies, agency-conducted laboratory studies, agency contractor studies, academic studies, and studies sponsored by nongovernmental organizations; *b*) conceal the identity of the chemical in question to minimize potential bias; *c*) have a number of experts score each study using the criteria; and then *d*) compile and compare the results to assess the degree of concordance among the assigned scores.

Unprecedented efforts will be devoted to toxicity testing and research in the coming years to meet growing public and regulatory demand for chemical safety information. It would be profoundly wasteful if all stakeholders do not act now to ensure that, when this information is developed, an effective system exists for assuring the public that the research is credible.

## Figures and Tables

**Table 1:** Criteria for assessing the credibility of scientific work.

Proposed criterion	BPC^a^	ILSI^b^	Henry and Conrad^c^	*IARC Code of Good Scientific Practice*^d^	FASEB Guidelines on “Conflict of Interest in Biomedical Research”^e^
1. Disclosure of funding sources and other competing interests	✓	✓ Scope limited to industry funding; other sources warrant thought	✓	✓	✓ Scope limited to industry funding

2. PI is legally guaranteed					
*a*) Freedom to publish	✓	✓ Must attempt to publish within some time frame	✓	✓ Duty to publish	✓
*b*) Authority to analyze and interpret results	—	~ ✓ PI must have control of statistical analysis	✓ PI must own data	—	~ ✓ PI must be involved in analysis and interpretation of results
*c*) Control of study design	✓	✓	—	—	—

3. Public release of data and methods	✓	~ ✓ PI and “appropriate auditors/reviewers” must have access to all data	✓ For data; does not address methods	~ ✓ Requests for use of data “to be encouraged”	~ ✓ PI should make reasonable efforts to provide data and materials to other investigators

4. Factual and transparent research objective and appropriate research design	—	✓	✓ Recognizes value of GLP [value of Common Rule noted (Conrad 2009)]	✓	✓

5. Peer review	✓	✓ “Mandatory prerequisite” for effectiveness of guidelines	✓	✓ Duty to publish in peer-reviewed journal	—

6. Prior listing in a public registry (where exists)	✓	—	—	—	—

7. No linkage of remuneration to outcome of experiment	—	✓	—	—	—

8. Disclosure of paid “name lending”	—	✓	—	—	—

9. Maintain clarity between CRO and academic auspices	—	✓	—	—	—

10. External review of research program	—	—	✓ (Conrad 2009)	—	—

Abbreviations: CRO, contract research organization; GLP, Good Laboratory Practice; PI, principal investigator. The check mark (✓) indicates complete or substantial adoption; the qualified check mark (~✓) indicates partial adoption; and the dash (—) indicates absence.

a BPC (2009).

b Rowe et al. (2009).

c Henry and Conrad (2008).

d IARC (2008).

e Brockway and Furcht (2006).
